# Proportion and characteristics of lacrimal drainage pathway disease and keratopathy in non-infectious corneal perforation using lacrimal syringing test

**DOI:** 10.1038/s41598-023-47248-9

**Published:** 2023-11-13

**Authors:** Sho Ishikawa, Takanori Sasaki, Takafumi Maruyama, Kei Shinoda

**Affiliations:** Department of Ophthalmology, Saitama Medical University, Saitama, Japan

**Keywords:** Diseases, Medical research, Signs and symptoms

## Abstract

Lacrimal drainage pathway disease-associated keratopathy (LDAK) has been associated with corneal perforation, which arises from both infectious and non-infectious corneal disorders. However, patients with corneal perforation are often not routinely tested for LDAK, and the potential risk posed by LDAK in the development of corneal ulcers has not been investigated in detail. This study aimed to assess the proportion and characteristics of LDAK in patients with non-infectious corneal perforation using lacrimal syringing test. This study enrolled 56 patients with corneal perforation treated at Saitama Medical University Hospital between January 2016 and September 2022. The causes of corneal perforation were trauma (n = 17, 30%), infection (n = 19, 34%), non-infection (n = 16, 29%), and unknown (n = 4, 7%). A lacrimal syringing test was performed on 12 patients with non-infectious corneal perforation and 4 with an unknown diagnosis. Among the 16 patients with non-infectious corneal perforation, 13 (81%) had lacrimal drainage disease, but only 3 (19%) patients had lacrimal puncta, as revealed by slit-lamp examinations. The primary bacterial species identified in lacrimal obstructive disease and lacrimal canaliculitis were *Staphylococcus* spp. and *Actinomycetes* spp. respectively. Lower temporal and peripheral corneal perforations were common. All patients underwent lacrimal surgery, and 6 (38%) were treated for corneal perforation without corneal surgery. Interestingly, several patients with LDAK who did not exhibit any lacrimal duct obstruction on slit-lamp examination. The study findings demonstrate the significance of the lacrimal syringing test for assessing LDAK in patients with corneal perforation, indicating LDAK as a potential cause of corneal perforation.

## Introduction

Corneal perforation arises as an emergency condition due to infectious and non-infectious corneal disorders. The causes of non-infectious corneal disorders include corneal melting after the removal of a metal foreign body, severe dry eye, lagophthalmos, oral anticancer drugs (ex. S-1), keratoconus, rheumatoid arthritis, neurotrophic ulcers, atopic keratoconjunctivitis, canaliculitis, and unknown causes, including Mooren’s ulcers^1^. Canaliculitis can lead to corneal perforation in non-infectious disorders, with a rarity of 8%^1^. Another study examined the causes of corneal perforation in 90 cases; however, it did not describe its association with lacrimal duct disease^2^. In contrast, 12 cases (13.3%) were listed as unknown^2^.

Some studies have reported that corneal perforations or ulcers are associated with lacrimal disorders, including lacrimal dacryocystitis^3,4^, and lacrimal canaliculitis^5^. Inoue et al.^6^ named the lacrimal drainage pathway disease-associated keratopathy (LDAK). Thus, LDAK is a potential risk factor for corneal ulcers. The primary findings suggestive of LDAK include a few cellular infiltrations at the site of ulceration and a large amount of discharge^6^. However, few reports exist, and the details are not yet available.

Among the 101 patients with unilateral lacrimal duct obstruction, 64 had contralateral asymptomatic lacrimal duct obstruction^7^. The patient was not tested for the lacrimal syringe pathway to detect lacrimal duct obstruction if no epiphora or eye discharge symptoms were observed. Therefore, LDAK may have been missed in some cases of corneal perforation. After our experience with a case of corneal perforation caused by canaliculitis in 2016^5^, we routinely performed a lacrimal syringing test in patients with corneal perforation.

In this study, we describe the results of the lacrimal syringing test and the proportion and characteristics of LDAK in patients with corneal perforation.

## Results

The causes of corneal perforation were classified as trauma (n = 17, 30%), infectious (n = 19, 34%), non-infectious (n = 16, 29%), and unknown (n = 4, 7%). The causes of non-infectious corneal perforation were corneal marginal ulcer (n = 5), corneal marginal ulcer with rheumatoid arthritis (n = 3), Sjogren’s syndrome (n = 4), lagophthalmos (n = 2), Mooren’s ulcer (n = 1), and side effects induced by anticancer drugs (n = 1) (Table 1). Two patients with corneal marginal ulcers and one with an unknown origin had lacrimal canaliculitis (punctal orifice swollen and red) on the first visit.

**Table 1:** Primary causes of corneal perforation.

Causes	Number of eyes (%)	Age (mean ± standard deviation)	Female/male
Trauma	17 (30%)	58.7 ± 23.1	5/11
Infection	19 (34%)	73.1 ± 14.7	6/13
Bacteria	10 (18%)	75.8 ± 13.8	6/4
Fungus	1 (2%)	87	1/0
Virus (Herpes virus)	6 (11%)	70.8 ± 9.87	4/2
Non-infectious	16 (29%)	75.8 ± 13.80	6/4
Corneal marginal ulcer	5 (9%)	80.0 ± 4.64	4/1
Sjogren syndrome	4 (7%)	79.8 ± 5.12	3/1
Rheumatoid arthritis	3 (5%)	80.0 ± 4.36	3/0
Lagophthalmos	2 (4%)	61.0 ± 15.60	1/1
Mooren’s ulcer	1 (2%)	50	0/1
Drug induced	1 (2%)	79	0/1
Unknown	4 (7%)	86.0 ± 13.1	3/1

A lacrimal syringing test was performed in 12 patients with non-infectious corneal perforation (excluded: two lagophthalmos, one drug-induced, and one Sjogren syndrome) and four patients with unknown diagnoses. On one Sjogren syndrome, we could not perform a lacrimal syringing test because the patient had received punctal plug occlusion therapy for severe dry eye. All patients showed little cellular inflammation in the ulcerated area on slit-lamp examination.

Table 2 shows the results of the lacrimal syringing test and patient characteristics. Before the lacrimal syringing test, three patients had the punctal orifice was swollen and red. Eight patients (50%) (two corneal marginal ulcers, one Sjogren syndrome, two rheumatoid arthritis, and three unknown patients) had a lacrimal obstruction in the lacrimal syringing test. The other eight patients (50%) (three with corneal marginal ulcers, two with Sjogren’s syndrome, one with rheumatoid arthritis, one with Mooren’s ulcer, and one unknown patient) did not have lacrimal obstructive disease; however, five (31%) patients (two with corneal marginal ulcers, one with Sjogren’s syndrome, one with rheumatoid arthritis, and one unknown patient) reflexed bacterial concretion during lacrimal syringing test. The lacrimal syringing tests revealed that LDAK was caused by nasolacrimal duct obstruction (8 cases) and lacrimal canaliculitis (5 cases). The average age of patients with LDAK was 82.5 ± 7.5 years (11 women and 2 men). The age of patients with LDAK was significantly higher than that of patients without LDAK (*p* = 0.008). Overall, four patients (25%) (two with corneal marginal ulcers, one with rheumatoid arthritis, and one with unknown diagnosis) had lacrimal pathway obstructions on the contralateral side.

**Table 2:** Results of the lacrimal syringing test and features of the patients.

Diagnosis	Age, sex	Locations of corneal perforation	Results of lacrimal syringing test	Result of culture
Corneal marginal ulcer	85, male	Peripheral, lower temporal	Obstruction with reflex discharge	None
	82, female	Peripheral, lower nasal	Obstruction without reflex discharge	None
	82, female	Peripheral, upper temporal	Passage with bacterial concretion	Actinomyces
	78, female	Peripheral, lower temporal	Passage with bacterial concretion	Actinomyces
	73, female	Peripheral, upper nasal	Passage	None
Sjogren syndrome	85, female	Paracentral, inferior	Passage with bacterial concretion	Actinomyces
	77, female	Central	Obstruction with reflex discharge	Achromobacter xylosoxidans
	74, female	Central	Passage	None
Rheumatoid arthritis	85, female	Paracentral, temporal	Passage with bacterial concretion	Actinomyces
	78, female	Central	Obstruction with reflex discharge	Staphylococcus epidermidis
	77, female	Paracentral, upper nasal	Obstruction with reflex discharge	Staphylococcus epidermidis (Methicillin-resistant)
Mooren’s ulcer	50, male	Peripheral, nasal	Passage	None
Unknown	105, male	Paracentral, lower nasal	Obstruction with reflex discharge	Staphylococcus aureus
	82, female	Central	Obstruction with reflex discharge	Staphylococcus constellatus
	82, female	Paracentral, temporal	Obstruction with reflex discharge	Pseudomonas aeruginosa
	75, female	Paracentral, inferior	Passage with bacterial concretion	Actinomyces

Seven of the eight (88%) patients in whom lacrimal obstruction with reflex discharge was detected using a lacrimal syringing test had bacteria in the bacterial culture. The bacterial culture results were as follows: two Staphylococcus epidermidis (two rheumatoid arthritis), one *Staphylococcus aureus* (one unknown), one *Staphylococcus constellatus* (one unknown), one *Achromobacter xylosoxidans* (one Sjogren syndrome), and one *Pseudomonas aeruginosa* (one unknown). Five patients who were discharged with bacterial concretion and a lacrimal syringing test detected *Actinomyces* on bacterial culture and histological pathology. Thirteen of the 16 (81%) patients with corneal perforation had lacrimal drainage disease. The locations of the corneal perforations were as follows: four central, six paracentral, and six peripheral. Among the patients with central corneal perforation, three (75%) had lacrimal drainage disease. Among the patients with paracentral corneal perforation, six (100%) had lacrimal drainage disease. Among patients with peripheral corneal perforation, four (67%) had lacrimal drainage disease. When the cornea was divided into four parts, three cases were located in the upper temporal, three were in the upper nasal, five were in the lower temporal, and six were in the lower nasal regions (including duplicates).

**Figure 1:**
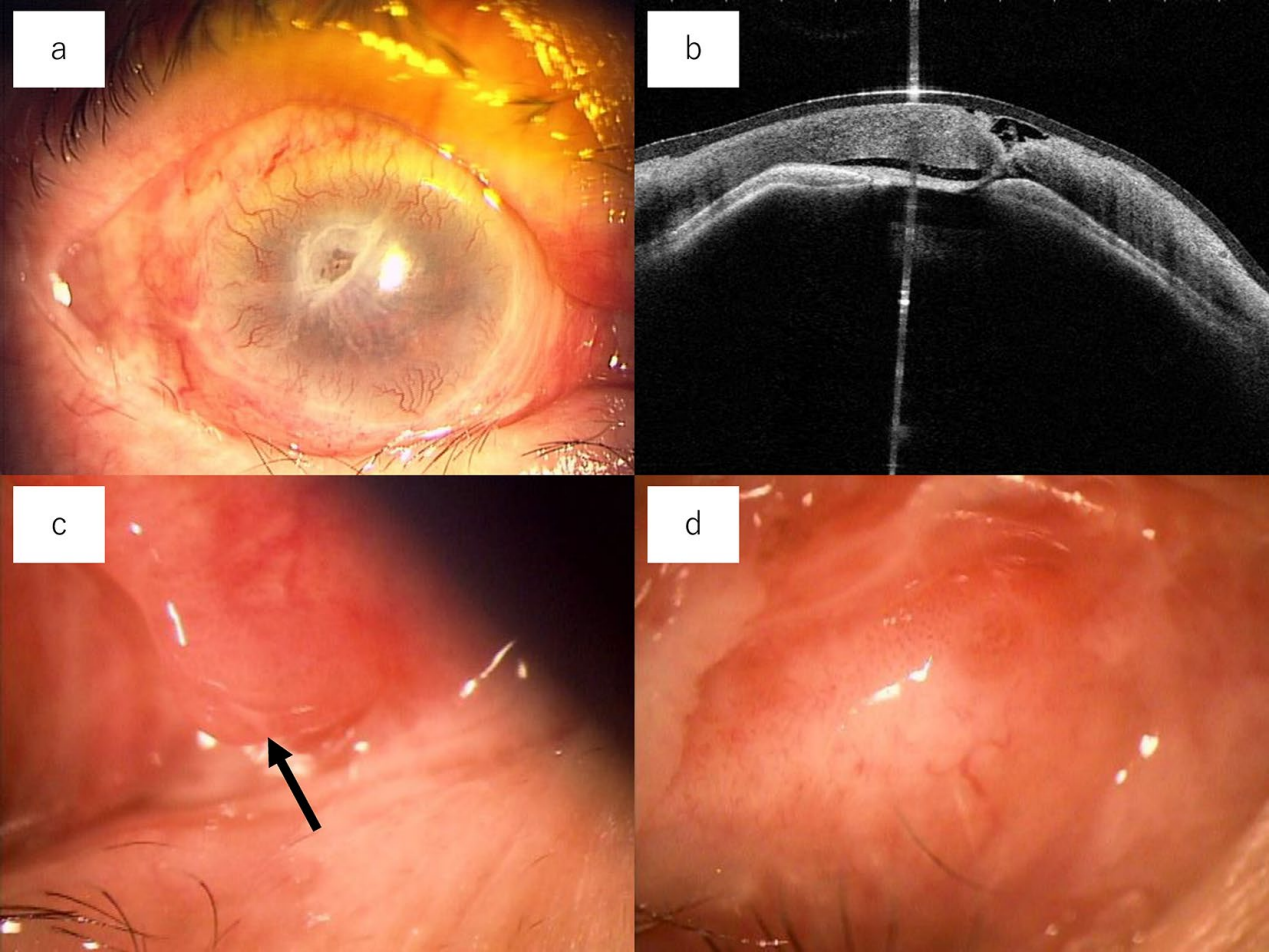
An 85-year-old female with a right corneal perforation associated with rheumatoid arthritis. (**a**) Corneal perforation was detected in the upper temporal region. Hyperemia was observed in the conjunctiva. (**b**) Anterior-segment optical coherence tomography. The anterior chamber disappeared despite the use of contact lenses. (**c**) Slight eye discharge (arrows) and redness were observed in the upper lacrimal puncta. (**d**) Redness was observed in the lower puncta and ocular discharge. In the lacrimal syringing test, saline did not pass through the nasal cavity or cause reflex discharge.

**Figure 2:**
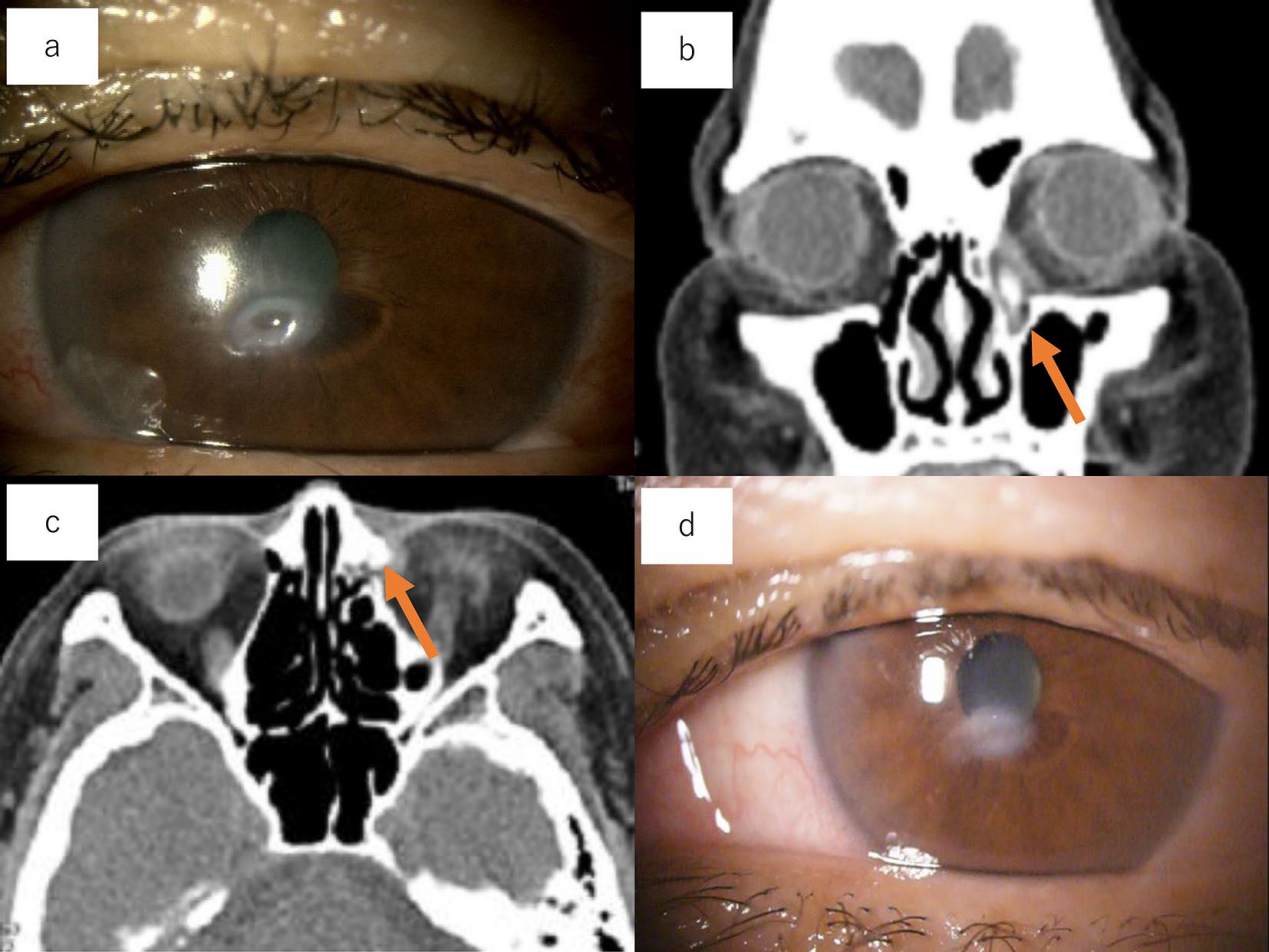
A 75-year-old female with left corneal perforation (unknown cause). (**a**) Corneal perforation was detected in the inferior left cornea. No lacrimal puncta findings were detected. (**b** and **c**) In the lacrimal syringing test, saline was passed through the nasal cavity and reflexed bacterial concretion. Bacterial concretions were detected in the lacrimal sac (arrows). (**d**) Seven days after lacrimal surgery, the corneal perforation disappeared, and corneal opacity decreased.

Table 3 shows the clinical course of the patients. Six patients were treated wearing contact lenses without surgery. Eight patients underwent localized keratoplasty, and two patients underwent eye removal. In contrast, nine patients underwent dacryocystectomy (DCT), three underwent stone curettage for lacrimal concretion, and two underwent lacrimal tube intubation. All surgical procedures were performed within 3 days of the diagnosis of corneal perforation. Eleven of the 16 (69%) patients were diagnosed with dry eye and treated. All DCT procedures were performed on patients with dry eyes and no symptoms of epiphora. Visual acuity improved in nine patients (two central, three paracentral, and four peripheral corneal perforations), worsened in four patients (two central, one paracentral, and one peripheral), and did not change in three patients (two paracentral and one peripheral). Postoperative visual acuity was changed from 0.03 ± 0.05 to 0.09 ± 0.14 in central corneal perforation, from 0.05 ± 0.06 to 0.06 ± 0.05 in paracentral corneal perforation, and from 0.29 ± 0.30 to 0.53 ± 0.38 in peripheral corneal perforation. More patients had better vision in the peripheral corneal perforation group than in the central (*p* = 0.027) and paracentral perforation groups (*p* = 0.016). No significant difference was observed between the central and paracentral perforation groups (*p* = 0.510). None of the patients experienced postoperative complications or corneal perforation recurrence for over a year. We presented two cases of LDAK. One patient had corneal perforation with lacrimal findings (Fig. 1), and the other had no lacrimal findings on slit-lamp examination (Fig. 2).

**Table 3:** Clinical course of patients.

Diagnosis	Age,sex	LDAK	Dryeye	Treatment for cornea	Treatment for lacrimal drainage	Visual acuity (pretreatment)	Visual acuity (posttreatment)	Location of corneal perforation
Corneal marginal ulcer	85, male	+	+	Contact lens	DCT	0.3	0.8	Peripheral
	82, female	+	+	LKP	DCT	0.15	0.1	Peripheral
	82, female	+	+	LKP	DCT	30 cm H.M	0.7	Peripheral
	78, female	+	−	Contact lens	stone curettage	0.7	0.8	Peripheral
	73, female	−	−	Eye removal	None	SL (−)	SL (−)	Peripheral
Sjogren syndrome	85, female	+	+	LKP	stone curettage	SL (+)	15 cm H.M	Paracentral
	77, female	+	+	Contact lens	DCT	0.02	0.02	Central
	74, female	−	+	LKP	None	0.1	0.3	Central
Rheumatoid arthritis	85, female	+	+	LKP	DCT	0.03	0.1	Paracentral
	78, female	+	+	LKP	DCT	20 cm H.M	20 cm H.M	Central
	77, female	+	+	Eye removal	DCT	10 cm H.M	SL (−)	Paracentral
Mooren’s ulcer	50, male	−	−	LKP	None	0.6	0.8	Peripheral
Unknown	105, male	+	+	Contact lens	DCT	0.15	0.07	Paracentral
	82, female	+	+	LKP	DCT	30 cm H.M	0.04	Central
	82, female	+	−	Contact lens	stone curettage LTI	0.1	0.1	Paracentral
	75, female	+	−	Contact lens	LTI	0.03	0.08	Paracentral

## Discussion

In this study, we showed that 13 of 16 (81%) patients with non-infectious and unknown corneal perforations had lacrimal drainage-associated diseases when using the lacrimal syringing test. Of the 13 patients with LDAK, six underwent corneal perforation without corneal surgery. Patients in the peripheral corneal perforation group had a significantly better visual acuity prognosis than those in the central and paracentral corneal perforation groups.

LDAK was first reported by Inoue et al.^6^. LDAK was defined as a non-infectious corneal ulcer (including corneal perforation) related to lacrimal drainage pathway disease. According to reports, LDAK indicated few cellular infiltrations of the ulcerated area, suggesting that the ulcers in LDAK are not caused by corneal inflammation^3,6,8^. The most common location of corneal perforation was the nasal or inferior peripheral location of the ulcers and copious ocular discharge. Chronic dacryocystitis and lacrimal canaliculitis cause lacrimal diseases^6^.

The causes of non-traumatic corneal perforation were divided into infectious (26%) and non-infectious (74%). The causes of non-infectious corneal perforation are severe dry eye (13%), lagophthalmos (13%), canaliculitis (9%), rheumatoid arthritis (4%), and unknown (22%)^1^. In this study, the cause of canaliculitis was 19%, which differs from that reported in a previous study. In this study, two patients with lacrimal canaliculitis were absent from the lacrimal plug findings on slit-lamp examination. When lacrimal pathway findings were absent, a lacrimal syringing test was not performed, which may have masked several undiagnosed LDAK cases. The cause of corneal perforation in 90 cases was not associated with lacrimal duct disease^2^.

In this study, 13 of the 16 (81%) patients with non-infectious and unknown corneal perforations had lacrimal drainage-associated diseases when using the lacrimal syringing test. Before the lacrimal syringing test, only three patients had lacrimal pathway findings, and the punctal orifice was swollen and red. This indicates that many cases of LDAK may be caused by the absence of lacrimal findings. Asymptomatic lacrimal pathway obstructions or stenosis also occurred on the contralateral side, ranging from 30 to 63% of cases^7,9^. These reports suggest that some cases of asymptomatic lacrimal drainage exist. Some reports suggest that LDAK is induced by toxins from bacteria that cause lacrimal drainage pathway diseases^1,3,5^. The relationship between the causative agents of lacrimal drainage pathway disease and LDAK needs to be investigated in a larger cohort. In this study, 11 of 16 patients were diagnosed with dry eye disease. Many cases of LDAK have been reported to be complicated by dry eye^6,8^. Dry eyes may mask symptoms of lacrimal duct disease, such as epiphora and purulent discharge, and lacrimal duct disease may thus be underdiagnosed^8^. Moreover, many cases of LDAK are associated with autoimmune diseases such as rheumatoid arthritis, Sjogren’s syndrome, systemic lupus erythematosus, and graft-versus-host disease^1,3–6,8^. Inflammatory cytokine levels in tears were elevated in rheumatoid arthritis^10^, Sjogren’s syndrome^11^, systemic lupus erythematosus^11,12^, and graft-versus-host disease^13^. However, most inflammatory cytokines were higher in the tears of the lacrimal obstructive disease group than in the normal group and rapidly decreased to normal levels after lacrimal surgical treatment^14^. Thus, elevated levels of tear cytokines may be involved in the development of LDAK.

In this study, four *Staphylococcus species*, one *Achromobacter xylosoxidans,* and one *Pseudomonas aeruginosa* were identified using a lacrimal syringing test and diagnosed as lacrimal duct obstructions because they did not pass through the nasal cavity. Epiphora and elevated tear meniscus height are usually observed in lacrimal duct obstruction, and purulent discharge is observed in lacrimal duct infections such as dacryocystitis. However, none of the patients in this study complained of epiphora. Presumably, dry eye masked the symptoms of epiphora. In contrast, *Actinomyces* was identified in five cases of lacrimal canaliculitis with a lacrimal syringing test and was diagnosed as canaliculitis because it passed through the nasal cavity. In a previous study, gram-positive bacteria were the most common cause of chronic dacryocystitis^15^, whereas aerobic gram-positive bacteria and *Actinomyces spp*. were the most common anaerobic bacteria and the cause of canaliculitis^16^. In this study, gram-positive bacteria were detected in four of the six cases of lacrimal obstruction, and *Actinomyces* were detected in all five cases of canaliculitis, consistent with previous reports. *Actinomyces* form proteolytic enzymes that may be associated with corneal ulceration^5,6^; therefore, *Actinomyces* infection may be a risk factor for corneal perforation. Although *Streptococcus* was not detected in this study, a previous study detected *Streptococcus* in four out of seven cases of LDAK with chronic dacryocystitis, indicating that *Streptococcus* should also be considered as a causative agent of LDAK.

The locations of the corneal perforations were the upper temporal (n = 3), upper nasal (n = 3), lower temporal (n = 5), and lower nasal (n = 6). The location of the corneal perforation was central in four cases, paracentral in six, and peripheral in six. Corneal perforation in LDAK can be located both paracentrally and peripherally, and this location of corneal perforation in LDAK may be one of the distinguishing feature between collagen diseases and autoimmune corneal ulcers, which typically manifest as peripheral ulcers. Consistent with previous reports, the most common location of corneal perforation in LDAK was the nasal or inferior peripheral location, some cases was occurred paracentral^6^. Visual acuity after surgical treatment improved in nine patients, worsened in four patients, and did not change in three patients in this study.

Regarding the location of the corneal perforation, visual acuity was significantly improved in peripheral perforations rather than in central and paracentral corneal perforations. Factors associated with poor visual acuity include non-traumatic corneal perforation and older age^2^. Treatment-resistant corneal ulcers complicated by chronic dacryocystitis can be controlled after lacrimal duct treatment^17^, and early diagnosis and planning for surgery are imperative in cases of lacrimal duct obstruction to manage corneal infection^15^. After treatment of lacrimal drainage pathway disease, patients show rapid healing of epithelial defects^6^. In this study, all patients were treated within three days; therefore, we considered that they had a relatively good visual acuity prognosis in the peripheral corneal perforation group. We attributed the difference in visual acuity prognosis to visual axis astigmatism induced by corneal transplantation, not to LDAK treatment.

This study had some limitations. First, this was a relatively small, retrospective, single-center case series. More extensive studies are needed for an in-depth characterization of the clinical features and diagnostic criteria of LDAK. Second, the lacrimal syringing test was performed only for non-infectious or unknown corneal perforations and not for traumatic or infectious corneal perforations. Perforations from infected corneal ulcers may also contain LDAK. Finally, the mechanisms underlying LDAK development remain unclear. Therefore, the possibility that peripheral ulcerative keratitis and other concealed causes may contribute to this condition and complicate lacrimal disease cannot be ruled out. Further investigations are required to elucidate these causes.

In conclusion, we demonstrated the results of the lacrimal syringing test and the proportion and characteristics of LDAK in patients with corneal perforation. We found that 13 of the 16 (81%) patients with LDAK did not have lacrimal puncta on slit-lamp examination. The findings indicate the importance of performing a lacrimal syringing test to assess LDAK in cases of corneal perforation, suggesting that LDAK may be a potential cause of corneal perforation.

## Materials and methods

### Participants

Institutional Review Board/Ethics Committee approval was obtained from the Ethics Committee of Saitama Medical University Hospital (2022-087). This study adhered to the tenets of the Declaration of Helsinki. The need for written informed consent was waived by the ethics committee of Saitama Medical University due to the retrospective design of the study.

We retrospectively enrolled 56 patients diagnosed with corneal perforation and treated at Saitama Medical University Hospital between January 2016 and September 2022. We categorized the causes of corneal perforation into three groups: trauma, infectious, and non-infectious. We routinely performed a lacrimal syringe pathway test for non-infectious corneal perforation in January 2016. In the non-infectious group, the demographic and clinical characteristics of the patients, including age, sex, systemic and ocular medical history, systemic and local predisposing factors, characteristics of the corneal ulcer (location, shape, and cellular infiltrations), results of the lacrimal syringe pathway, culture from discharge, and treatment method, were collected from medical records. In this study, we diagnosed infectious corneal perforation based on the following criteria: (1) presence of corneal infiltration on slit-lamp examination and (2) positive bacterial or fungal culture in the corneal infiltration sites or positive PCR of the virus in the tear film. Non-infectious corneal perforations were defined as those that did not meet the criteria for traumatic or infectious corneal perforation. All infectious and non-infectious patients were examined using blood tests, electrocardiography, and chest and abdominal radiography to determine the presence of collagen disease and rheumatoid arthritis. All non-infectious patients were examined with a laser flare meter (FM 600-a®, KOWA, Japan) to determine uveitis.

The location of the corneal perforation was divided into three regions: central (within a 2 mm zone from the corneal center), paracentral (surrounding a 2–6 mm annulus), and peripheral. The peripheral regions of the corneal perforation were further divided into the superior, inferior, nasal, and temporal regions.

We used a 23-gauge Nakamura’s lacrimal washing single-sized needle (Inami, Tokyo, Japan) filled with saline solution. The lacrimal pathway was washed to determine obstruction. Lacrimal obstructive disease was diagnosed when saline solution did not reach the nasal cavity. We cultured the reflex discharge from the lacrimal pathway after washing it. A seed swab no. 3 (Cygni Medical, Tokyo, Japan) was used to wipe the reflex discharge and collect specimens. When we detected bacterial concretions from the reflex discharge, we performed both culture and histopathological examinations.

### Statistical analyses

All statistical analyses were performed using JMP version 17® software (SAS Institute, Tokyo, Japan). All data are expressed as means ± standard deviation. The Mann–Whitney U test was used to compare the ages of patients with and without LDAK. The Mann–Whitney U test was used to compare the visual acuity of central, paracentral, and peripheral corneal perforations. All analyses of the objective findings used values from the right eye. Statistical significance was set at *p* < 0.05 and *p* values < 0.001 were presented as *p* < 0.001.

## Data Availability

The datasets generated during and/or analysed during the current study are available from the corresponding author on reasonable request.
